# Low income as a determinant of exercise capacity in COPD

**DOI:** 10.1177/1479972318809491

**Published:** 2018-11-18

**Authors:** Ana Sofia Porta, Nyanjok Lam, Paul Novotny, Roberto Benzo, the NETT Research Group

**Affiliations:** 1 Mindful Breathing Laboratory, Division of Pulmonary and Critical Care Medicine, Mayo Clinic, MN, USA; 2 Health Sciences Research-Biomedical Statistics and Informatics, Mayo Clinic, MN, USA

**Keywords:** COPD, social determinants of health, exercise capacity, quality of life, pulmonary rehabilitation, CPET, 6-minute walking test

## Abstract

Exercise capacity (EC) is a critical outcome in chronic obstructive lung disease (chronic obstructive pulmonary disease (COPD)). It measures the impact of the disease and the effect of specific interventions like pulmonary rehabilitation (PR). EC determines COPD prognosis and is associated with health-care utilization and quality of life. Field walking tests and cardiopulmonary exercise test (CPET) are two ways to measure EC. The 6-minute walking test (6MWT) is the commonest and easiest field test. CPET has the advantage of assessing maximal aerobic capacity. Determinants of EC include age, gender, breathlessness, and lung function. Previous research suggests that socioeconomic status (SES), a meaningful factor in COPD, may also be associated with EC. However, those findings have not been replicated. We aimed to determine whether SES is an independent factor associated with EC in COPD. For this analysis, we used the National Emphysema Treatment Trial (NETT) database. NETT was a multicenter clinical trial where severe COPD patients were randomized to lung volume reduction surgery or medical therapy. Measures used were taken at baseline, postrehabilitation. Patients self-reported their income and were divided in two groups whether it was less or above US$30,000. Patients with a lower income had worse results in 6MWT (*p* < 0.0001). We found an independent association between income and the 6MWT in patients with severe COPD after adjusting for age, gender, lung function, dyspnea, and living conditions (*p* < 0.0007). One previous publication stated the relationship between income and EC. Our research confirms and extends previous publications associating EC with income by studying a large and well characterized cohort of severe COPD patients, also addressing EC by two different methods (maximal watts and 6MWT). Our results highlight the importance of addressing social determinants of health such as income when assessing COPD patients.

## Introduction

Exercise capacity (EC) is a critical outcome in chronic obstructive lung disease (chronic obstructive pulmonary disease (COPD)).^1^ It measures the impact of the disease and tests the effect of specific interventions.^1–3^ EC determines COPD prognosis due to its relation with hospitalizations for exacerbations and all-cause mortality.^4,5^ Importantly, from a patient-centered focus, it is related to health-care utilization, quality of life (QoL), and symptoms.^3^

Field walking tests and cardiopulmonary exercise test (CPET) are two ways to measure EC. Among those, the 6-minute walking test (6MWT) is the commonest and easiest.^1,2,6^ CPET has the advantage of assessing maximal aerobic capacity and contributes better to the differential diagnosis.^7^ EC determinants are many,^6,8^ including age, breathlessness, and lung function.

Socioeconomic status (SES), a meaningful factor in COPD related to QoL,^9^ physical activity,^10^ completion of pulmonary rehabilitation (PR), and all-cause mortality,^11^ may also predict EC. Recent data show that SES also influences EC, with lower income patients having poorer results^12^; however, that finding has never been replicated.

Our aim is to determine whether income is an independent factor associated with EC in COPD, which would raise its importance as a meaningful factor to be assessed.

## Methods

The present analysis used the National Emphysema Treatment Trial (NETT) database.^13^ This multicenter trial randomized severe COPD patients with no significant comorbidities to lung volume reduction surgery or medical therapy, after 6–10-week PR.^13,14^ Smoking cessation, biochemically validated, was required at least 6 months before randomization. Patients were divided in two groups whether their annual reported income was less or above US$30,000, a limit chosen following data of US census bureau and Pew Research center.^15,16^

EC was measured by the 6MWT and maximal watts during CPET, following the American Thoracic Society Guidelines (ATS).^6,17^ Pulmonary function tests followed ATS.^18,19^

Statistical analyses, associations between income and categorical variables, were tested between income groups using χ^2^tests. Associations between income level and continuous variables were tested between groups using Wilcoxon tests. The association between income and 6MWT as well as income and CPET was explored using linear models adjusted for marital status, loneliness, age, gender, FEV1% predicted, modified medical research council scale (MMRC), smoking pack years, and education. All analyses were tested with 5% type I error rates with no adjustments for multiple testing. Analyses were carried out using SAS version 9.4.

## Results

In total, 1218 patients completed PR and were analyzed using baseline data. Table 1 shows population’s characteristics and Tables 2 and 3 show the models designed to determine the independent association between income and EC.

**Table 1.** Characteristics of all 1206 patients at baseline^a^.

Characteristics	Income ≥US$30,000 (*N* = 563)	Income ≤US$30,000 (*N* = 643)	*p* Value
Medical treatment	52.0%	49.1%	0.3153
Sex, male	65.4%	57.5%	*0.0054*
Age, mean (SD) (years)	67.2 (5.5)	65.6 (6.6)	*0.0001*
Married	83.3%	48.5%	*<0.0001*
Lonely^b^	5.5%	10.1%	*0.0031*
Smoking pack years	65.4 (29.3)	63.6 (32.6)	0.0826
Post BD FEV1% pred, mean (SD)	26.8 (7.1)	26.6 (7.2)	0.3947
6MWD in feet, mean (SD)	1250.7 (314.8)	1171.7 (306.8)	*<0.0001*
Maximum load (watts), mean (SD)^c^	41.6 (22.7)	36.8 (20.4)	*0.0001*
Total days in hospital, mean (SD)	3.0 (7.8)	3.3 (7.6)	0.3840
Any days in hospital, yes	31.7%	33.8%	0.4620
Total visits to ER, mean (SD)	0.6 (1.1)	0.7 (1.3)	0.5015
Any visit to ER, yes	37.5%	39.7%	0.4659
Follow-up status, dead^d^	39.4%	41.7%	0.4278
MRC Dyspnea Scale			*0.0058*
0	1.4%	1.6%	
1	0.2%	0.5%	
2	29.2%	22.1%	
3	23.5%	19.9%	
4	45.7%	55.9%	
Education beyond high school	62.9%	35.1%	<0.0001

*N*: sample; SD: standard deviation; Post BD FEV1% pred: postbronchodilator forced expiratory volume in 1 second % predicted; ER: emergency department; MRC scale: Medical Research Council Dyspnea Scale; 6MWD: 6-minute walk distance. Every value in italics <0.05 denotes significance (standard practice).

^a^ Base-line measurements were obtained after rehabilitation but before randomization.

^b^Loneliness was measured by asking subjects “Do you feel lonely or socially isolated now or in the last 3 days?” An answer was either “yes” or “no.”^20^

^c^ Maximum load (watts) was chosen to measure exercise capacity during CPET.

^d^ The follow-up period was 2 years.

**Table 2.** Income as a determinant of 6MWT, adjusted for age, sex, Post FEV1% predicted, MMRC, loneliness, married status, smoking pack years, and education beyond high school.

Variables	DF	Parameterestimates	Standard error	*t* Value	Pr > |*t*|
Intercept	1	1607.40	95.58	16.82	<0.0001
Married	1	27.47	18.45	1.49	0.1367
Lonely or Isolated^a^	1	−23.24	29.50	−0.79	0.4310
*Income < US$30,000*	1	*−48.15*	17.76	−2.71	*0.0068*
Age	1	−10.87	1.35	−8.05	*<0.0001*
Sex, male	1	180.85	17.82	10.15	*<0.0001*
Post BD FEV1% pred	1	16.10	1.17	13.75	*<0.0001*
MMRC	1	−67.59	8.61	−7.85	*<0.0001*
Education beyond high school^b^	1	32.89	16.78	1.96	0.0502
Smoking pack years	1	−0.11	0.26	−0.42	0.6720

DF: degrees of freedom; Pr > |*t*|: *t*-test significance level; Post BD FEV1% pred: post bronchodilator forced expiratory volume in 1 second; MMRC: Modified medical research council scale. Every value in italics <0.05 denotes significance (standard practice).

^a^Loneliness and isolation was measured by asking subjects “Do you feel lonely or socially isolated now or in the last 3 days?” An answer was either “yes” or “no.”^20^

^b^Education was determined by dividing patients into two groups: those who had education levels beyond high school and those who did not.

**Table 3.** Income as a determinant of watts in CPET, adjusted for age, sex, Post FEV1% predicted, mMRC, loneliness, married status, smoking pack years and education beyond high school.

Variables	DF	Parameter estimates	Standard error	*t* Value	Pr > |*t*|
Intercept	1	48.24	5.99	8.06	<0.0001
Married	1	1.79	1.16	1.55	0.1222
Lonely or isolated^a^	1	−0.48	1.85	−0.26	0.7957
*Income < US$30,000*	1	*−2.27*	1.11	−2.04	*0.0419*
Age	1	−0.77	0.08	−9.14	*<.0001*
Sex, male	1	22.14	1.12	19.83	*<0.0001*
Post BD FEV1% predicted	1	1.36	0.07	18.60	*<0.0001*
MMRC	1	−2.88	0.54	−5.34	*<0.0001*
Education beyond high school^b^	1	2.14	1.05	2.04	*0.0417*
Smoking pack years	1	0.01	0.02	0.47	0.6416

DF: degrees of freedom; Pr > |*t*|: *t*-test significance level; Post BD FEV1% pred: postbronchodilator forced expiratory volume in 1 second; MMRC: Modified medical research council scale. Every value in italics <0.05 denotes significance (standard practice).

^a^Loneliness and isolation was measured by asking subjects “Do you feel lonely or socially isolated now or in the last 3 days?”. An answer was either “yes” or “no.”^20^

^b^Education was determined by dividing patients into two groups: those who had education levels beyond high school and those who did not.

The association between 6MWT and income (Table 2) remained significant after adjusting for meaningful confounders such as sex, age, Post BD FEV1% predicted, MMRC, marriage, loneliness, smoking pack years, and education beyond high school (yes or no). We also found a significant association between income and maximal load in watts on CPET (Table 3) validating the 6MWT results. In addition, our results showed individuals with low income found themselves lonelier (Tables 1 and 2).

## Discussion

We found that low income is associated with worse results in the 6MWT after adjusting for meaningful confounders. The latter was further validated using another measure of EC: watts in CPET. Our findings confirm and extend a previous report^12^ but in a larger and more characterized cohort of severe COPD, using two different EC measures.

Given the 6MWT’s prognostic significance,^4–6^ our findings are important because usually income is not taken into account in COPD evaluation, despite being a factor that is easily obtainable, and conveys information about patients’ overall social condition. Plausible explanations for our results might include that financial constrains may limit patients’ access to programs like PR, as stated by previous publications.^21–23^ The fact of finding low-income participants more lonely suggests another social, yet critical repercussion of low income. Loneliness is independently related to poorer outcomes and higher health-care utilization in COPD (article in press).

### Limitations

Given that NETT selected severe emphysema COPD patients without significant comorbidities, our results may limit generalizability.

## Conclusions

In severe COPD patients, low income is independently associated with EC. Our results may be of importance to fully assess patients with severe COPD addressing social determinants of health.

## References

[1] ZuWallack RL Haggerty MC Jones P Clinically meaningful outcomes in patients with chronic obstructive pulmonary disease. Am J Med 2004; 117(suppl 12A): 49S–59S. 10.1016/j.amjmed.2004.10.020 15693643

[2] Solway S Brooks D Lacasse Y A qualitative systematic overview of the measurement properties of functional walk tests used in the cardiorespiratory domain. Chest 2001; 119(1): 256–270. 11157613 10.1378/chest.119.1.256

[3] Cazzola M MacNee W Martinez FJ Outcomes for COPD pharmacological trials: from lung function to biomarkers. Eur Respir J 2008;31(2): 416–469. 18238951 10.1183/09031936.00099306

[4] Durheim MT Smith PJ Babyak MA Six-minute-walk distance and accelerometry predict outcomes in chronic obstructive pulmonary disease independent of Global Initiative for Chronic Obstructive Lung Disease 2011 Group. Ann Am Thorac Soc 2015; 12(3): 349–356. 25568929 10.1513/AnnalsATS.201408-365OC PMC4418313

[5] Pinto-Plata VM Cote C Cabral H The 6-min walk distance: change over time and value as a predictor of survival in severe COPD. Eur Respir J 2004; 23(1): 28–33. 14738227 10.1183/09031936.03.00034603

[6] ATS Committee on Proficiency Standards for Clinical Pulmonary Function Laboratories. ATS statement: guidelines for the six-minute walk test. Am J Respir Crit Care Med 2002; 166(1): 111–117. 12091180 10.1164/ajrccm.166.1.at1102

[7] Salzman SH The 6-min walk test: clinical and research role, technique, coding, and reimbursement. Chest 2009; 135(5): 1345–1352. 19420202 10.1378/chest.07-1682

[8] Foglio K Carone M Pagani M Physiological and symptom determinants of exercise performance in patients with chronic airway obstruction. Respir Med 2000; 94(3): 256–263. 10783937 10.1053/rmed.1999.0734

[9] Bak-Drabik K Ziora D The impact of socioeconomic status on the quality of life in patients with chronic obstructive pulmonary disease. Pneumonol Alergol Pol 2010; 78(1): 3–13. 20162513

[10] Gimeno-Santos E Frei A Steurer-Stey C Determinants and outcomes of physical activity in patients with COPD: a systematic review. Thorax 2014; 69(8): 731–739. 24558112 10.1136/thoraxjnl-2013-204763 PMC4112490

[11] Cho KH Nam CM Lee EJ Effects of individual and neighborhood socioeconomic status on the risk of all-cause mortality in chronic obstructive pulmonary disease: a nationwide population-based cohort study, 2002-2013. Respir Med 2016; 114: 9–17. 27109806 10.1016/j.rmed.2016.03.003

[12] Eisner MD Blanc PD Omachi TA Socioeconomic status, race and COPD health outcomes. J Epidemiol Community Health 2011; 65(1): 26–34. 19854747 10.1136/jech.2009.089722 PMC3017471

[13] Fishman A Martinez F Naunheim K A randomized trial comparing lung-volume-reduction surgery with medical therapy for severe emphysema. N Engl J Med 2003; 348(21): 2059–2073. 12759479 10.1056/NEJMoa030287

[14] Rationale and design of The National Emphysema Treatment Trial: a prospective randomized trial of lung volume reduction surgery. The National Emphysema Treatment Trial Research Group. Chest 1999; 116(6): 1750–1761. 10593802 10.1378/chest.116.6.1750

[15] U.S. Census Bureau. Current population reports, P60-213, Money income in the United States: 2000. Washington, DC: U.S. Government Printing Office, 2001.

[16] Pew Research Center analysis of the 2000 decennial census and 2014 American Community Survey (IPUMS). America’s shrinking middle class: A close look at changes within metropolitan areas. Washington, DC: Pew Research Center, 2016.

[17] American Thoracic Society; American College of Chest Physicians. ATS/ACCP Statement on cardiopulmonary exercise testing. Am J Respir Crit Care Med 2003; 167(2): 211–277. 12524257 10.1164/rccm.167.2.211

[18] Miller MR Hankinson J Brusasco V Standardisation of spirometry. Eur Respir J 2005; 26(2): 319–338. 16055882 10.1183/09031936.05.00034805

[19] Miller MR Crapo R Hankinson J General considerations for lung function testing. Eur Respir J 2005; 26(1): 153–161. 15994402 10.1183/09031936.05.00034505

[20] Kaplan RM Anderson JP Wu AW The quality of well-being scale. applications in AIDS, cystic fibrosis, and arthritis. Med Care 1989; 27(suppl 3): S27–S43. 2921885 10.1097/00005650-198903001-00003

[21] Thorpe O Johnston K Kumar S Barriers and enablers to physical activity participation in patients with COPD: a systematic review. J Cardiopulm Rehabil Prev 2012; 32(6): 359–369. 22941449 10.1097/HCR.0b013e318262d7df

[22] Amorim PB Stelmach R Carvalho CR Barriers associated with reduced physical activity in COPD patients. J Bras Pneumol 2014; 40(5): 504–512. 25410838 10.1590/S1806-37132014000500006 PMC4263331

[23] Steiner MC Lowe D Beckford K Socioeconomic deprivation and the outcome of pulmonary rehabilitation in England and Wales. Thorax 2017; 72(6): 530–537. 28077613 10.1136/thoraxjnl-2016-209376 PMC5520271

